# Scalable optical vortex arrays enabled by the decomposition of Laguerre–Gaussian beams into three Hermite–Gaussian modes and multibeam interference

**DOI:** 10.1038/s41377-026-02254-0

**Published:** 2026-04-08

**Authors:** Yoshiki Nakata, Noriaki Miyanaga, Yuki Kosaka, Masataka Yoshida

**Affiliations:** 1 Institute of Laser Engineering, The University of Osaka, Suita, Osaka Japan; 2 Institute for Laser Technology, Nishi-ku, Osaka Japan; 3 JGC Holdings, Yokohama-shi, Kanagawa Japan; 4 Osaka Gas Co., Ltd., Chuo-ku, Osaka Japan

**Keywords:** Laser material processing, Optical techniques, Lasers, LEDs and light sources

## Abstract

Optical vortex arrays (OVAs) are a promising platform for massively parallel photonics, but existing generation methods are limited by low power capacity, restricted scalability, or high complexity. We demonstrate a simple and robust method that overcomes these limitations by combining a reformulation of Hermite–Gaussian to Laguerre–Gaussian mode conversion representation with multibeam interference. Using a compact diffractive optical element (DOE)–spiral phase plate (SPP)–$4f$ Fourier optical system, we experimentally generated a triangular lattice of 3070 coherent vortices with a peak power of 58 megawatts (MW). This result demonstrates more than three orders of magnitude improvement in both the vortex number and peak power compared with those of spatial light modulator (SLM)-, metasurface-, and conventional DOE-based OVA systems, establishing a new benchmark for large-scale, high-power vortex array generation. Moreover, our method provides OVA generation with high peak and average power while remaining inherently scalable in the vortex number, wavelength, and input laser power owing to the interference-based DOE–SPP design. This capability not only advances fundamental optical vortex science but also provides a powerful route for applications in parallel laser processing, broadband chiral photonics, massively parallel biophotonics, and future explorations in quantum and nonlinear photonics.

## Abstract (web summary)

Decomposition of Laguerre–Gaussian beams into three Hermite–Gaussian modes enables interference-based generation of large-scale optical vortex arrays, experimentally demonstrated using a simple DOE–SPP $4f$ optical system.

## Graphical abstract

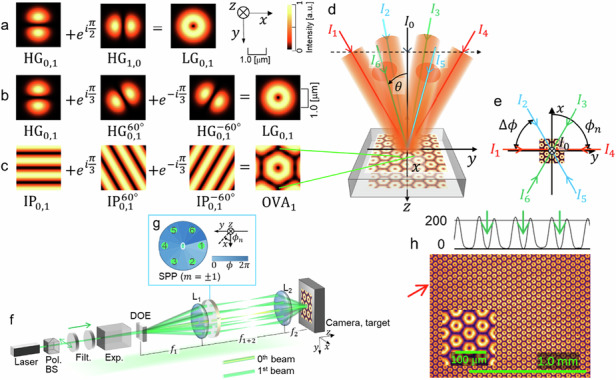

## Introduction

The history of light field control began in the 19th century with the discovery of interference patterns^1^, which led to the identification of optical vortices (OVs) as phase singularities in wavefields and the development of higher-order modes, such as the Hermite–Gaussian (HG) and Laguerre–Gaussian (LG) modes. A milestone was reached in the 1990s with the discovery of orbital angular momentum (OAM) in the LG mode^2,3^. This mode, characterized by a spiral wavefront and a central singularity, opened new avenues for superresolution microscopy^4^, chiral crystallization^5^, optical manipulation^6^, and microstructure fabrication^7^.

Since then, this concept has been extended to optical vortex arrays (OVAs), which allow parallel processing and new functionalities, including biological and colloidal particle sorting^8^. However, conventional OVA generation methods are subject to fundamental limitations. Spatial light modulator (SLM)-based dynamic holography—computer-generated holograms (CGHs) displayed on liquid-crystal SLMs^8–10^—offers reconfigurability but is fundamentally power-limited and typically produces tens of vortices at subwatt powers^9^. In contrast, static diffractive optical elements (DOEs) fabricated on solid substrates^11^, such as fused-silica phase plates, provide higher stability and damage resistance but are nonreconfigurable and limited in scalability. Metasurfaces also enable compact OVA generation^12^, but they are similarly restricted in terms of power handling and flexibility.

Interference-based approaches developed before the advent of lasers are promising alternatives, as they impose no theoretical upper limit on the number of patterns^1^. Nevertheless, the generation of spiral interference patterns at high power and on a large scale has not been demonstrated experimentally until now. Here, we revisit LG mode formation and present a reformulated three-mode decomposition of LG beams in terms of HG modes—the first such revision in 30 years. Instead of representing LG modes as the sum of two HG modes with a $\pi /2$ phase shift^2,3^, we present them as the sum of three HG modes, each rotated by 60°. Integrating this with a multibeam interference framework enables the generation of vortex wavefronts with effectively unlimited vortex numbers. We have demonstrated this experimentally using a robust, high-power $4f$ optical system incorporating a single spiral phase plate (SPP) to generate a triangular array of 3070 phase-coherent OVs at a peak power of 58 MW. This performance exceeds that of the previous methods by more than three orders of magnitude. This system facilitates direct laser processing of chiral microstructures and provides experimental evidence of OAM transfer in OVAs. This methodology paves the way for new research into superparallel chiral phenomena, as well as quantum and nonlinear chiral science.

## Results

### HG‒LG mode decomposition and interference-based framework

Here, we review the LG and HG modes, discuss their conventional conversion, and present a reformulation of the mode conversion representation that enables explicit integration with interference. LG and HG modes are solutions to the Helmholtz equation in cylindrical and Cartesian coordinates, respectively. The LG mode, denoted ${{\rm{LG}}}_{p,l}$, has a spiral wavefront characterized by a radial index $p\ge 0$ and topological charge $l$, which sets the central phase singularity and OAM per photon ($l{\rm{\hslash }}$, where ${\rm{\hslash }}$ is the reduced Planck constant). The HG modes (${{\rm{HG}}}_{n,m}$) use Hermite polynomials ${H}_{n}\left(x\right)$^13^ and orders $n$ and $m$ along the $x$ and $y$ axes.

To validate the classical conversion of HG-to-LG modes, we analyse the superposition of the ${{\rm{HG}}}_{0,1}$ and ${{\rm{HG}}}_{1,0}$ modes at 90° with a phase difference of ${\rm{\pi }}/2$. This generates the ${{\rm{LG}}}_{0,1}$ mode:

1 $${E}_{0,1}^{\rm{LG}}\propto {E}_{0,1}^{\rm{HG}}+{e}^{i\frac{\pi }{2}}{E}_{1,0}^{\rm{HG}}$$

The light intensity is given by:

2 $$I\propto \int {\left|{E}_{0,1}^{{\rm{LG}}}\right|}^{2}{\rm{dt}}$$

**Fig. 1:**
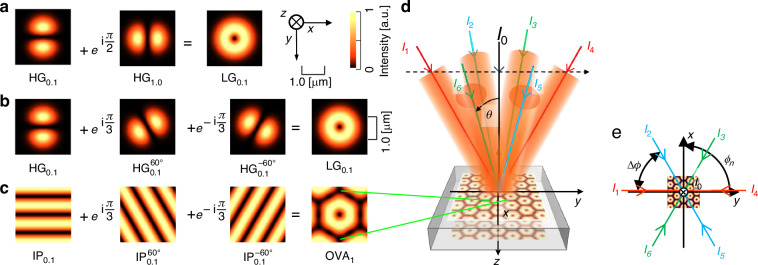
Conceptual mode conversions for optical vortex (OV) and optical vortex array (OVA) formation. **a** Superposition of Hermite–Gaussian modes ${{\rm{HG}}}_{\mathrm{0,1}}$ and ${{\rm{HG}}}_{1,0}$ with a phase difference of ${\rm{\pi }}/2$ yields the Laguerre–Gaussian ${{\rm{LG}}}_{0,1}$ mode. **b** Superposition of three ${{\rm{HG}}}_{0,1}$ modes rotated by 0°, +60°, and −60° (phases 0, +${\rm{\pi }}/3$, and $-{\rm{\pi }}/3$) produces the ${{\rm{LG}}}_{0,1}$ mode. **c** Superposition of three two-beam interference patterns (${\mathrm{IP}}_{0,1}$, ${\mathrm{IP}}_{0,1}^{60^\circ }$, and ${\mathrm{IP}}_{0,1}^{-60^\circ }$) with phase shifts of 0, $+{\rm{\pi }}/3$, $-{\rm{\pi }}/3$ generates the OVA₁ mode. **d** Three-dimensional schematic of the six-beam interference geometry. The polar angle $\theta$ is defined relative to the $+z$ axis (beam-propagation direction). **e** Top-view schematic showing the azimuthal angles ${\phi }_{n}$ and their increments $\Delta \phi =60^\circ$. Opposing beam pairs corresponding to the three ${{\rm{IP}}}_{0,1}$ modes are color coded: ${I}_{1}-{I}_{4}$ (red), ${I}_{2}\,-\,{I}_{5}$ (blue), and ${I}_{3}\,-\,{I}_{6}$ (green). The undiffracted zeroth-order beam ${I}_{0}$ is coaxial with the $+z$ axis. Images: numerical simulations (Wolfram Mathematica)

For visualization, we use λ = 532 nm and a beam waist of 0.6 μm (Fig. 1a).

However, conventional HG-to-LG mode conversion representation is not suitable for large-scale OVA generation via interference, as will be discussed later. Here, we demonstrate that ${{\rm{LG}}}_{0,1}$ can be expressed as a combination of three rotated HG modes. The conventional HG-to-LG mode conversion representation is based on the relationship between the Laguerre and Hermite polynomials:

3 $$\left(x+{iy}\right)\,{L}_{0}^{1}\left({r}^{2}\right)=\,{H}_{1}\left(x\right){H}_{0}\left(y\right)+i{H}_{0}\left(x\right){H}_{1}\left(y\right)$$

where ${H}_{n}\left(x\right)$ and ${L}_{p}^{l}$ denote the Hermite and Laguerre polynomials^2^, respectively. This relationship leads to conventional HG-to-LG mode conversion (Eq. (1)).

We generalize this by rotating ${{\rm{HG}}}_{0,1}$ by an angle $\theta$ (see Supplementary Information Section 1):

4 $${H}_{0}\left(x\cos \theta -y\sin \theta \right){H}_{1}\left(x\sin \theta +{\rm{ycos}}\theta \right)\propto {H}_{1}\left(x\right){H}_{0}\left(y\right)\sin \theta +{H}_{0}\left(x\right){H}_{1}\left(y\right)\cos \theta$$

Thus, ${{\rm{LG}}}_{0,1}$ can be represented as a coherent sum of three ${{\rm{HG}}}_{0,1}$ modes at $\theta =0,\,\pm 60^\circ$, with phase differences of $0,\,\pi /3,{\rm{and}}-\pi /3$:

5 $${E}_{0,1}^{{\rm{LG}}}\propto {E}_{0,1}^{{\rm{HG}}}+{e}^{i\frac{\pi }{3}}{E}_{0,1}^{{\rm{HG}}}\left({60}^{^\circ }\right)\,+{e}^{-i\frac{\pi }{3}}{E}_{0,1}^{{\rm{HG}}}\left(-{60}^{^\circ }\right)$$

Equations (1) and (5) both originate from Eq. (3) and yield identical intensity distributions, as illustrated in Fig. 1a, b.

To clarify the geometric correspondence between the rotated HG modes and the equivalent beam interference representation, we introduce additional schematic illustrations. Figure 1d provides a three-dimensional overview of the six-beam interference geometry, illustrating how the polar angle $\theta$ is defined relative to the $+z$ axis. We adopt a right-handed coordinate system in which the $+z$ axis corresponds to the beam-propagation direction. Figure 1e shows a top-view representation in which the azimuthal angles ${\phi }_{n}$ and their increments $\Delta \phi =60^\circ$ (${\phi }_{n+1}-{\phi }_{n}=\Delta \phi =2\pi /N$, $N=6$) are explicitly indicated. The opposing beam pairs that form the three ${{IP}}_{\mathrm{0,1}}$ modes are color-coded for clarity (${I}_{1}-{I}_{4}$ in red, ${I}_{2}\,-\,{I}_{5}$ in blue, and ${I}_{3}\,-\,{I}_{6}$ in green). The undiffracted zeroth-order beam ${I}_{0}$ is coaxial with the $+z$ axis. This schematic provides an intuitive understanding of the multibeam interference geometry that generates the ${{\rm{OVA}}}_{1}$ mode. This reformulation enables integration with a multibeam interference scheme for OVA generation. The three rotated HG modes in Eq. (5) can be replaced by three two-beam interference patterns (IP modes), as follows.

**Fig. 2:**
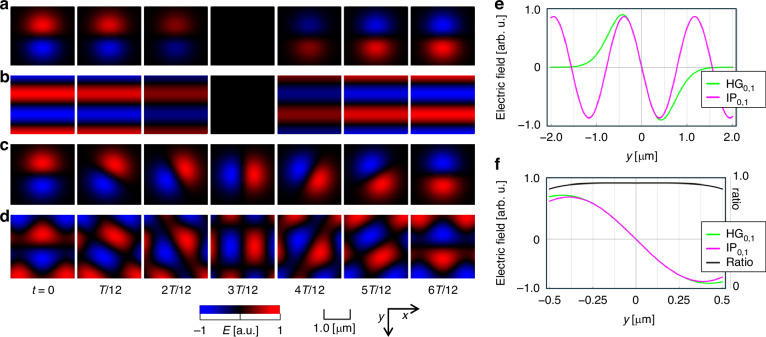
Simulated electric field distribution of key modes and their cross-section along the *y*-axis at *t=0.* **a**–**d** Field distributions for the **a** ${{\rm{HG}}}_{\mathrm{0,1}}$, **b** ${{\rm{IP}}}_{0,1}$, **c** ${{\rm{LG}}}_{0,1}$, and **d** ${{\rm{OVA}}}_{1}$ modes. The color represents normalized intensity. **e**, **f** Cross-sectional profiles along the $y$-axis at $t=0$ for (**a**, **b**). $T$: time in one optical cycle

To verify their equivalence, we numerically simulated their fields for both the HG and IP modes ($\lambda =532$ nm). Figure 2a shows the simulated electric field ${E}_{0,1}^{{\rm{HG}}}$ (product of the ${{\rm{TEM}}}_{00}$ and Hermite polynomials) at the half-period $T/2={\rm{c}}/2\lambda$, revealing a blinking two-lobe pattern^14^ where the electric field polarity alternates periodically.

The corresponding IP mode is as follows^1^:

6 $${E}_{n}={E}_{n0}{e}^{i\left[{k}_{n}\left(-x\sin {\theta }_{n}\cos {\phi }_{n}-y\sin {\theta }_{n}\sin {\phi }_{n}+z\cos {\theta }_{n}\right)-{\omega }_{n}t+{\alpha }_{n}\right]}$$

where ${E}_{n0}$ is the amplitude, ${k}_{n}=2\pi /{\lambda }_{n}$, ${\theta }_{n}$ is the angle relative to the $z$-axis, ${\phi }_{n}$ is the azimuthal angle, ${\omega }_{n}$ is the angular frequency, and ${\alpha }_{n}$ is the phase shift (for symmetry, ${\theta }_{n}=\theta =20^\circ$ and ${k}_{n}=k$). Next, considering the substitution of the three rotated HG modes in Eq. (5) with three IP modes, six-beam interference consisting of three pairs, each forming an IP mode (Fig. 1c, d), is assumed: *I*_*1*_ & *I*_*4*_, *I*_*2*_ & *I*_*5*_, and *I*_*3*_, & *I*_*6*_. The beams were arranged with an azimuthal angle difference of $\Delta \phi =60^\circ$ and a phase difference of $\Delta \alpha =\pi /3$.

We simulated the IP mode ${E}_{0,1}^{{\rm{IP}}}$ for the opposing beam pair ${E}_{1}+{E}_{4}$ on the $y$-axis (${\phi }_{1}=90^\circ$, ${\alpha }_{1}=0\,{and}\,{\alpha }_{4}=\pi$, Fig. 2b), showing a blinking stripe pattern in which the electric field polarity alternates, similar to ${E}_{0,1}^{{\rm{HG}}}$ in Fig. 2a. A direct comparison along the y-axis and $t=0$ (see Fig. 2e, f) reveals that the field ratio ${E}_{0,1}^{{\rm{IP}}}/{E}_{0,1}^{{\rm{HG}}}$ is consistent within $\pm$0.035% for $y\le 0.20$ μm, confirming their precise equivalence near the origin. Furthermore, both fields vanish along the $x$-axis.

Using the validated equivalence and Eq. (5), we express the coherent combination of three symmetric IP modes as follows:

7 $$\sum {E}_{\Delta \alpha =\pi /3}^{\mathrm{IP}}={E}_{0,1}^{\mathrm{IP}}+{e}^{i\frac{\pi }{3}}{E}_{0,1}^{\mathrm{IP}}\left({60}^{^\circ }\right)+{e}^{-i\frac{\pi }{3}}{E}_{0,1}^{\mathrm{IP}}\left({-60}^{^\circ }\right)$$

as illustrated in Fig. 1c. For clarity and reproducibility, the phase and azimuthal angles assigned to each interference beam can be written in generalized form as follows:

$${\alpha }_{n}=\left(n-1\right)\frac{\pi }{3},{\,\phi }_{n}={\phi }_{0}+(n-1)\frac{2\pi }{6}$$

where *ϕ*_0_ = 90° and $n=1,\,2,\ldots ,\,6$. These expressions yield the increments $\Delta \alpha =\pi /3$ and $\Delta \phi ={60}^{\circ }=\pi /3$ used in the OVA configuration and correspond directly to the values shown in Eq. (7). The spiral wavefront and OVA intensity (from temporal integration) and their similarity to the LG mode are discussed below.

We simulated the fields for ${E}_{0,1}^{{\rm LG}}$ and $\sum {E}_{\Delta \alpha =\pi /3}^{\mathrm{IP}}$ (Eqs. (1) and (7)). Both simulations show two-lobed patterns with field polarity reversal after half a rotation (see Fig. 2c, d). These results provide the first direct visualization of the spiral wavefronts with $l=1$ for both modes as time-evolving fields. Reversing $\triangle \alpha$ switches the rotation direction, yielding $l=-1$ (see Fig. S1c, d in the Supplementary Information). The six-beam geometry creates an infinite, coherent, triangular lobe arrangement^15^ (Fig. S2a). We refer to this as the “OVA mode” ${{\rm{OVA}}}_{l}$, which has a topological charge of $l=6\Delta \alpha /2\pi =\pm 1$.

**Fig. 3:**
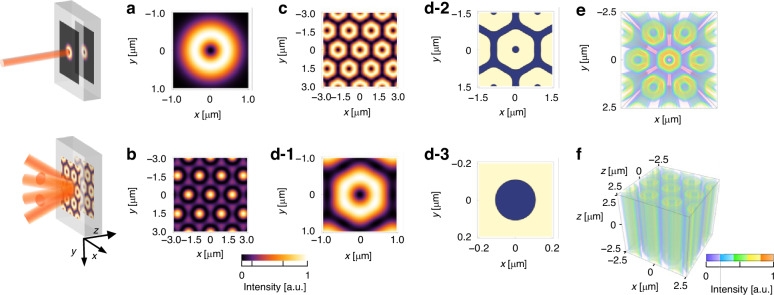
Schematics and simulated intensity patterns for optical vortices (OVs) and optical vortex arrays (OVAs). Left: conceptual schematics of a single OV (top) and a single OVA (bottom). **a** Intensity distribution of the ${{\rm{LG}}}_{0,1}$ mode. **b** OVA₀ mode ($\Delta \alpha =0$). **c** OVA₁ mode ($\Delta \alpha =\pi /3$). **d-1** Magnified view of the central region of the OVA₁ mode, showing the vortex singularity and its surrounding intensity distribution. **d-2** 20% threshold contour of the OVA₁ intensity within the region of interest (ROI), highlighting the rounded-hexagonal envelope and the relative positions of neighboring OVs arising from the intrinsic sixfold symmetry of the interference field. **d-3** Further magnified 20% threshold contour at the vortex center, demonstrating that the central singularity remains nearly circular and is analogous to a single optical vortex. **d-2**, **d-3** regions above the 20% threshold are shown in light yellow, whereas lower-intensity regions are shown in dark blue. **e**, **f** 3D views of the OVA₁ mode (different angles). All images were obtained by numerical integration of the electric field over one optical cycle $T$

The simulated intensity distributions for ${{\rm{OVA}}}_{0}$ ($\Delta \alpha =0$) and ${{\rm{OVA}}}_{1}$ at $z=0$ were calculated by integrating ${\left|\sum {E}_{\Delta \alpha }^{\mathrm{IP}}\right|}^{2}$ over one cycle $T$, along with ${{\rm{LG}}}_{0,1}$, as shown in Fig. 3a–c. The center of ${{\rm{OVA}}}_{1}$ is magnified in Fig. 3d-1. These results confirm that the singularities distinctly appear in both ${{\rm{LG}}}_{0,1}$ and ${{\rm{OVA}}}_{1}$. The singularities of the OVs in ${{\rm{OVA}}}_{1}$ form a triangular lattice with the following coordinates:

8 $$\left(x,y\right)=\left({\it a\varLambda} +{\rm{\it b}}\frac{1}{2}{\it \varLambda} ,{\it b}\frac{\sqrt{3}}{2}{\it \varLambda} \right),a,b=0,\pm 1,\pm 2\ldots$$

with ${\it \varLambda} =4\pi /(\sqrt{3}k\sin \theta )$ (see Supplementary Information 2).

To gain insight into the possible optical origin of the radial features later observed in the ablation patterns, we further analyzed the thresholded intensity distribution of the ${{\rm{OVA}}}_{1}$ mode. As shown in Fig. 3d-2, the 20% threshold contour within the region of interest reveals a rounded-hexagonal envelope together with the relative positions of neighboring OVs, reflecting the intrinsic sixfold symmetry of the underlying six-beam interference field. Moreover, a further magnified view of the threshold contour at the vortex center (Fig. 3d-3) demonstrates that the vortex core itself remains nearly circular and closely resembles the intensity minimum area of a single OV.

The 3D images in Fig. 3c (Fig. 3e, f) confirm that the OVs in ${{\rm{OVA}}}_{1}$ have linear singularities extending along the correlation region, with a constant diameter and period^16^, unlike conventional OVs in free space.

In a previous examination of two-IP modes with an azimuthal difference of $\Delta \phi ={\rm{\pi }}/2$, corresponding to conventional two-mode HG decomposition, it was shown that the resulting interference field does not produce a circular OVA but instead yields phase singularities arranged on a square lattice. To clarify this point in the present context, we explicitly examined the corresponding two-IP configurations with $\Delta \alpha ={\rm{\pi }}/2$, as shown in Supplementary Fig. S3. Consistent with the previous analysis, we found that the singularities form a square lattice, but no donut pattern appears^17,18^. While this geometry is useful in multipoint superresolution microscopy^19^, it lacks rotational symmetry for well-defined OAM, underscoring the advantage of the OVA formed by three IP modes.

### Experimental verification of the OVA and its intensity singularities

**Fig. 4:**
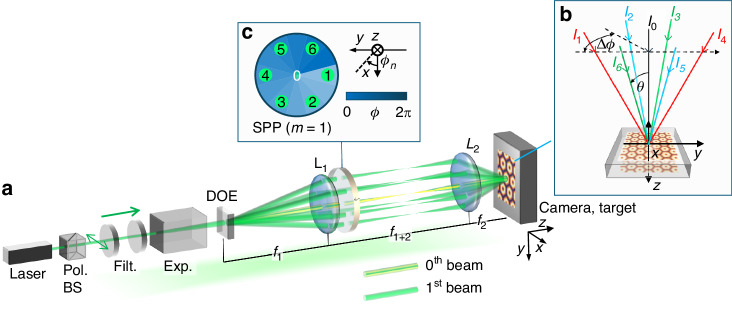
Experimental configuration for OVA formation using a *4f* optical system. **a** Schematic: laser source, polarization beam splitter (Pol. BS), neutral-density filter (Filt.), beam expander (Exp.), diffractive optical element (DOE; Holo/Or Ltd., Ness Ziona, Israel, 61% diffraction efficiency at 532 nm), convex lenses (L1, L2), and spiral phase plate (SPP; Holo/Or Ltd.), positioned ~50 mm before the Fourier plane towards L1. **b** $4f$ system output plane for the camera or target position. **c** Beam incidence on the SPP, where the adjacent beams’ azimuthal difference is $\Delta \phi =$60°, ensuring a phase shift of $\Delta \alpha =\pi /3$ for OVA₁ formation

**Fig. 5:**
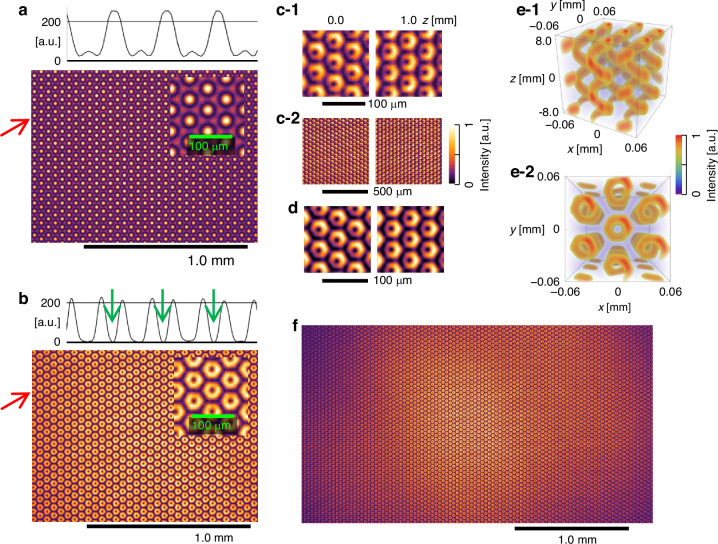
Experimental OVA intensity patterns and interference with a reference wave obtained using a 532-nm CW laser diode (LD). **a** OVA₀ mode ($\Delta \alpha =0$): hexagonal array, no singularities. **b** OVA₁ mode ($\Delta \alpha =\pi /3$): donut patterns, singularities marked (green arrows); insets in (**a**, **b**) are magnified images, and the graphs above show the cross-sectional intensity (red arrows). **c-1**, **c-2** Interference patterns for OVA_1_ and the planar reference wave observed at different $z$. **d** Simulated pattern matching (**c-1**) parameters. **e-1**, **e-2** 3D representations of (**d**), highlighting spiral structures. **f** Uncropped image corresponding to (**b**). From this image, ~3070 vortices were identified within the camera’s field of view; additional vortices extending beyond the recorded area indicate that the actual number is slightly greater. Images: CMOS camera (DMK 72AUC02, Imaging Source, Germany)

**Fig. 6:**
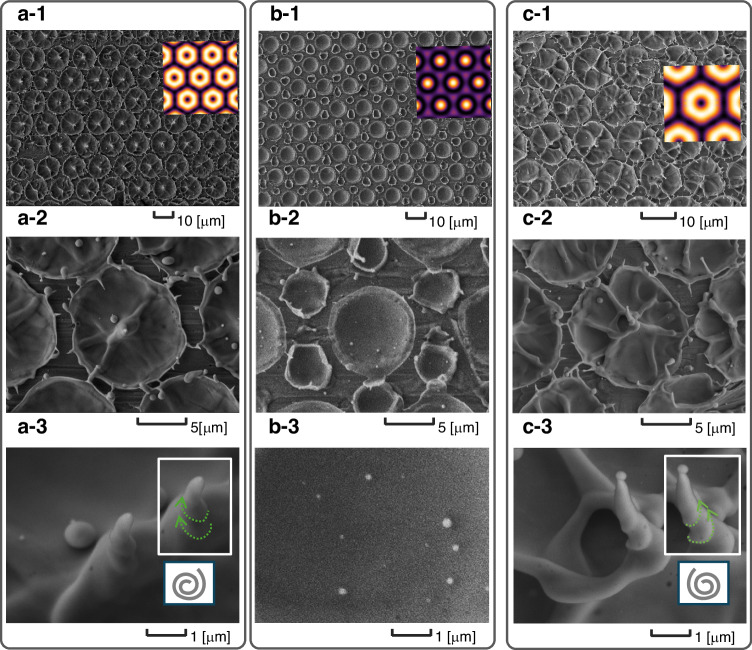
Copper (Cu) target ablation using OVA modes generated by the second harmonic of a nanosecond Nd:YAG laser at 532 nm. Cu targets processed with **a-1**, **a-2**, and **a-3** OVA₁ mode; **b-1**, **b-2**, and **b-3** OVA₀ mode; **c-1**, **c-2**, and **c-3** OVA₋₁ mode. **a-1**, **b-1**, and **c-1** show low-magnification views of the processed areas, where the overall spatial arrangement of the ablation patterns can be observed. The periodic ablation patterns are consistent with the simulated OVA lattice overlaid on the images. **a-2**, **b-2**, **c-2** show magnified views of representative regions. **a-3**, **b-3**, and **c-3** show high-magnification images. In particular, **a-3**, **c-3** show locally chiral nanoneedle structures formed at singularities under the OVA₁ and OVA₋₁ modes, respectively. To facilitate visual interpretation of the handed morphology, the chiral structures in these panels are extracted and overlaid onto the original images, with auxiliary guidelines (green dashed arrows) added to highlight the handed features. OVA₁ yields donut-like patterns and chiral nanoneedles at singularities; OVA₀ does not. The chiral direction in OVA₁ and OVA₋₁ reflects the topological charge $l$. This process resulted in a shot energy of 28.8 mJ, a peak power of 5.76 MW, and an average fluence of 1.43 J cm^−2^. The lattice constant was 13.1 μm, and the shot energy per vortex was 6.38 μJ. Compared with single OV ablation at 1064 nm^36^, the per-vortex energy was reduced by three orders of magnitude

We implemented the proposed principle using a robust, high-power $4f$ Fourier optical system, which is illustrated schematically in Fig. 4a. This consisted of a DOE to produce six first-order beams and two convex lenses to form the $4f$ system and correlate the beams (see Fig. 4b), as well as an SPP to impose a phase shift of $\Delta {\rm{\alpha }}$. For this setup, we drew on our experience with $4f$ optical systems for interference pattern control and material processing^15,16,20^. The laser source was either a linearly polarized 532-nm continuous-wave (CW) diode laser (LD, used in the experiments shown in Fig. 5) or the second harmonic of a nanosecond Nd:YAG laser (Nd:YAG SHG, used in the experiments shown in Fig. 6). Excluding the laser, the setup occupied an area of ~180 × 500 mm² on the optical table.

In Fig. 4b, the six beams produced by the DOE are arranged in a hexagonal configuration and correlated through the $4f$ system so that they overlap at the output plane. This configuration allows for stable interference between the six beams and establishes the triangular lattice geometry of the OVA.

In Fig. 4c, the SPP is inserted concentrically with the six-beam array. By introducing an additional phase shift of $\Delta {\rm{\alpha }}={\rm{\pi }}/3$, the OVA₁ mode is generated, in which each vortex in the triangular lattice carries a uniform topological charge. Reversing the SPP produced a phase shift of $\Delta {\rm{\alpha }}=-{\rm{\pi }}/3$ and formed the OVA₋₁ mode. To match the resolution of the imaging system, the interference angle was set to $\theta =0.72^\circ$, resulting in a lattice period of ${{\it \varLambda}} =48.8$ μm.

Using the LD source, Fig. 5a shows the intensity distribution of the OVA₀ mode ($\Delta {\rm{\alpha }}=0$), which formed a regular triangular lattice without the SPP. With the SPP inserted ($\Delta {\rm{\alpha }}={\rm{\pi }}/3$), the OVA₁ mode was generated (Fig. 5b), forming a triangular lattice of donut-shaped beams containing singularities. Cross-sectional intensity analysis revealed multiple singularities, indicated by the green arrows in Fig. 5b, in good agreement with the numerical simulations. From the untrimmed image corresponding to Fig. 5b (Fig. 5f), the total number of vortices was determined to be ~3070, which far exceeds the vortex counts reported in previous OVA studies^8,10,12,21–24^.

To verify the spiral wavefront of the OVA, interference experiments were performed using a planar reference wave, in accordance with established methods for characterizing OVs and their phase singularities^25,26^. Such plane-wave interference techniques have been widely used to directly visualize azimuthal phase structures and to identify the topological charge of OVs.

The resulting crescent-shaped interference fringes (Fig. 5c, d), recorded at $z=0$ mm and $z=1.0$ mm, respectively, demonstrate that all OVs carry phase singularities with a uniform topological charge of $l=1$. The crescent patterns rotate along the propagation direction at a rate of 52.9 degrees per millimeter. This rotation arises from the longitudinal phase mismatch between the vortex field and the on-axis planar reference wave, whose axial phase advance differs by a factor of $\cos {\theta }_{n}$. Accordingly, the rotation of the interference pattern can equivalently be described in terms of a helical pitch, which is given by $p=2\pi /\Delta {k}_{z}$, where $\Delta {k}_{z}=k(1-\cos {\theta }_{n})$. For ${\theta }_{n}=0.72^\circ$, this relation yields a pitch of $p=6.7$ mm, which is consistent with the experimentally observed rotation rate.

The simulated three-dimensional interference fields (Fig. 5e1, e2) consistently exhibit tornado-like standing-wave intensity structures. These structures do not indicate physical twisting or rotation of the optical field itself but instead result from interference between waves with different longitudinal phase advances. This interference converts the azimuthal phase structure of the OVs into a helical intensity distribution extending along the $z$-axis (Fig. 5e).

We tested the functionality of the system in high-power material processing experiments using an Nd:YAG SHG source. A copper surface was irradiated with OVA₁, resulting in the formation of periodic ablation spots with a characteristic donut-shaped pattern (Fig. 6a-1, as well as chiral nanoneedles at the vortex singularities (Fig. 6a-2, 6a-3). In contrast, OVA₀ produced no such structures (see Fig. 6b-2, 6b-3), which demonstrates that the observed nanostructures originated from the transfer of OAM. The twist direction depended on $l$ (see Fig. 6a-3, 6c-3), which is consistent with the transfer of OAM. Notably, the sixfold radial features and the formation of chiral nanostructures are not observed uniformly at every lattice site. This variability is attributed primarily to local inhomogeneities of the mechanically polished target surface, which can influence the ablation and resolidification dynamics. Consistent with this interpretation, prior studies have reported that vortex-driven ablation can produce needle-like features without an unambiguous macroscopic twist depending on the irradiation and material conditions^27^. This process achieved a shot energy of 28.8 mJ, a peak power of 5.76 MW, and an average fluence of 1.43 J cm^−2^. The shot-to-shot energy fluctuation of the Nd:YAG SHG source was 3.5% (manufacturer specification). The lattice constant was 13.1 μm, and the shot energy per vortex was 6.38 μJ.

Compared with ablation by a single OV using a nanosecond pulse laser at a wavelength of 1064 nm^28^, our approach reduced the energy requirement per OV by three orders of magnitude because of the higher absorptivity of copper at 532 nm and the smaller spot size (${{Reflectivity}}_{(532,\,1064{nm})}$ = 0.602 and 0.973, respectively). These results provide direct evidence that the demonstrated MW OVA can drive chiral light–matter interactions under high-power conditions.

## Discussion

The goal of this study was to demonstrate a scalable, high-power OVA and to explore its practical applications in parallel photonics, chiral science, and quantum technology. Using three rotationally symmetric IP modes in a high-power $4f$ optical setup with an SPP, we realized a megawatt (MW)-class coherent OVA system and experimentally verified large-scale OVA generation, phase singularities, and OAM.

With respect to peak power, our scheme is compatible with Ti:sapphire femtosecond lasers. For instance, a 785-nm commercial laser (Coherent Astrella, 9 mJ, 100 fs) combined with a DOE (77% efficiency) and optics (98% transmittance) can produce estimated OVA peak powers of up to 65 gigawatts. Our compact system surpasses SLM-based OVAs (tens of OVs at $\sim 0.6$ W)^9^ and metasurface converters (40 mW)^12^, establishing a new regime for quantum, nonlinear, chiral, and ultrafast laser processing applications.

In quantitative terms, our DOE–SPP $4f$ system produces >3000 vortices at 58 MW. This contrasts sharply with the tens of vortices generated by SLM-based approaches at 0.6 W and the limited vortex arrays obtained with metasurfaces at tens of milliwatts. This simultaneous improvement in both the vortex number and power represents an improvement of more than three orders of magnitude.

Table 1 summarizes representative methods for OVA generation and highlights their respective advantages and limitations in terms of the number of vortices, power capacity, and reconfigurability. For completeness, DMD-based holographic approaches are also included^29,30^, which typically generate a single structured beam rather than large-scale vortex arrays. SLM-based dynamic holography offers high reconfigurability, allowing for arbitrary and adaptive control of the vortex position and number. However, it has limited power handling and low throughput. In contrast, static DOEs offer greater stability and resistance to damage but lack reconfigurability. Metasurfaces enable compact integration but sacrifice both power capacity and tunability. Our static DOE–SPP $4f$ system combines the scalability of interference methods with MW-class power handling.

**Table 1:** Comparisons of this work with previously reported methods

Method	Typical vortex number	Peak/average power capacity	Reconfigurability	References
SLM-based dynamic holography (CGH on SLMs)	tens	$\sim 0.6$ W (CW, 532 nm)	high	^8–10^
DMD-based holography	~1	low	high	^29,30^
Static DOEs (fused silica)	hundreds	$\sim$W	none	^11^
metasurfaces	tens	$\sim 40$ mW (CW)	none	^12^
This work: DOE–SPP $4f$ system	3070	58 MW (ns pulse, 532 nm)	static but scalable	this work

The total angular momentum is controllable in our system. For an OVA with $\Delta {\rm{\alpha }}=0,\pm {\rm{\pi }}/3$, the OAM is $l=0,\pm 1$. With no polarizer after the DOE, the spin angular momentum (SAM) is $s=0,\pm 1$. This yields a total angular momentum of $J=l+s=0,\pm 1,\pm 2$, as with conventional OVA generation. The OVA preserves the amplitude, wavelength, polarization, and SAM of the input beam, supporting versatile data acquisition and advanced data science applications. Furthermore, since all six beams originate from the same DOE, their relative phases are intrinsically stable; the OVA pattern remained unchanged even when the setup was lightly vibrated or when individual optics were touched, demonstrating the high robustness of the configuration without active phase control. Finally, in the untrimmed OVA image (Fig. 5f), the reduced intensity of the outer vortices arises from the finite Gaussian envelope of the input beam and, predominantly, from the limited active area of the CMOS camera, which truncates the outer part of the interference field.

Scalability is another hallmark. When a 25 mm focal length lens is used, the maximum interference angle is ${\theta }_{n}=26.9^\circ$^31^, yielding a period of ${\it \varLambda} =4\pi /(\sqrt{3}k\sin \theta )=1.36$ μm at 532 nm and up to 208,000 OVs per mm²—~1000 times greater than that achieved with SLM- or metasurface-based methods.

In summary, this robust and flexible configuration enables OVA generation with unprecedented vortex number and power, achieving more than three orders of magnitude improvement over conventional SLM- and metasurface-based systems. It establishes a new benchmark for large-scale, high-power vortex array generation while remaining inherently scalable in the vortex number, wavelength, and input laser power through interference-based DOE–SPP design. Furthermore, this capability facilitates direct laser processing of chiral microstructures and provides experimental evidence of OAM transfer in OVAs. In the future, this method will open pathways to parallel laser processing, broadband chiral photonics, massively parallel biophotonics, and new explorations in quantum and nonlinear photonics, as well as extensions to other waves such as electron beams^32,33^ and neutrons^34,35^. In this study, our analysis and experiments are intentionally limited to the cases of $l=\pm 1$ (and the corresponding ${{\rm{OVA}}}_{0}$ mode with $l=0$), which represent the fundamental building blocks for large-scale OVAs. Whether the present interference-based framework can be extended to higher-order LG modes or to radial orders $p > 0$ has not yet been systematically examined, and such generalizations therefore remain outside the scope of the current work. Exploring these possibilities represents an interesting direction for future research.

## Materials and methods

### Simulation

The primary aim of the simulations was twofold: to validate analytical expectations regarding OVA formation and intensity singularities. All the simulations were performed using Wolfram Mathematica (version 14.2, Wolfram Research). To simulate interference patterns, the intensity distribution $\sum {\left|E\right|}^{2}$ was integrated over one full oscillation period, $\Delta t=\lambda /c$, where $\lambda$ is the wavelength and $c$ is the speed of light. For the temporal electric field intensity distributions shown in Fig. 3, the sum of the electric fields, $\sum E$, was directly calculated.

To simulate interference between the OVA and a reference plane wave, we used an intensity ratio of the first-order beam (${E}_{1{\rm{st}}}$) to the zeroth-order beam (${E}_{0{\rm{th}}}$) of $\sqrt{{I}_{1{\rm{st}}}}:\sqrt{{I}_{0{\rm{th}}}}=1:1.17$. This ratio was experimentally determined by comparing the intensities of the zeroth- and first-order beams generated by the DOE.

### Experimental setup

To experimentally verify the generation of the OVA and its intensity singularities, as well as to demonstrate a robust and straightforward high-power OVA configuration, we implemented a tabletop optical setup based on Fourier optics, employing a $4f$ configuration (Fig. 4a). We employed a horizontally polarized CW laser diode (CPS532, Thorlabs, Newton, NJ, USA) operating at $\lambda =532.0$ nm and an SHG Nd:YAG pulsed laser (Surelite I, Continuum, Milpitas, CA, USA). The 532 nm wavelength was selected because of its established use in previous OV formation experiments. The input to the DOE was a base Gaussian beam, and no additional beam expansion stage was used because the intrinsic beam quality and wavefront flatness of the laser sources were sufficient for generating stable first-order diffracted beams.

A DOE (Holo/Or Ltd., Ness Ziona, Israel) with a diffraction efficiency of 61% at 532 nm was used to split the input beam into six first-order diffracted beams. These were separated by 60° azimuthal angles around the zeroth-order beam and had equal power. The higher-order beams and the zeroth-order beam were blocked by an iris diaphragm to isolate the first-order beams. The intensity ratio between the first- and zeroth-order beams was experimentally determined as $\bar{{I}_{1{\rm{st}}}}:\bar{{I}_{0{\rm{th}}}}=1:1.38$.

Beam interference at the surface of the camera or copper target was achieved via a $4f$ optical system consisting of two convex lenses (L1 and L2). Various combinations of focal lengths (${f}_{1}$ = 100 mm or 200 mm and *f*_*2*_ =400 mm, 200 mm, or 30 mm) were selected to adjust the lattice period ($\varLambda$). Imaging was performed using a 5-MP CMOS camera with a pixel size of 2.2 μm (DMK 72AUC02, Image Source Co., Ltd., Bremen, Germany). Note that the high-power OVA (period of 13.1 μm) could not be directly resolved because of this pixel size, and only the low-power OVA was imaged. These experiments were conducted at room temperature under atmospheric conditions, with no special environmental controls. The image data were directly captured and analyzed using the standard image processing tools provided by the manufacturer and Windows 365 PowerPoint.

An SPP, designed to impose a helical phase front corresponding to a topological charge of $l=1$ at 532 nm, was coaxially aligned with the zeroth-order beam. This SPP, which is a standard product purchased from Holo/Or Ltd., was placed ~50 mm towards the L1 lens side relative to the Fourier plane of the $4f$ optical system. The DOE precisely sets the difference in the azimuthal angle ($\Delta \phi$) between the beams to 60°, ensuring a fixed phase shift ($\Delta \alpha$) of $\pi /3$ between the beams. Thus, the topological charge of the OVA remained consistent. The exact number of phase steps of the SPP was not specified by the vendor, but commercially available SPPs typically employ tens of discrete phase levels, and the resulting phase quantization error is small compared with the required π/3 phase steps for OVA formation. Reversing the orientation of the SPP resulted in a phase shift of $\Delta \alpha =-\pi /3$, forming the OVA₋₁ mode. The specifications of the DOE and the SPP are summarized in Tables 2 and 3. Detailed specifications of the DOE and the SPP can be found in Tables S1 and S2 in the Supplementary Information.

**Table 2:** Specifications of the DOE (MS609RYX, Holo/Or Ltd.)

Number of spots	6 (diffracted) +1 (zero-order)
Power uniformity	Approximately equal among six beams
Azimuthal spacing	${60}^{^\circ }$ increments
Full diffraction angle [deg.]	1.31 at 488 nm, 1.43 at 532 nm
Overall efficiency	~77% at 488 nm (manufacturer specification), 61% at 532 nm (measured)

**Table 3:** Specifications of the spiral phase plate (VL-209-QYA, Holo/Or Ltd.)

Designed wavelength [nm]	532
Topological charge	1

## Supplementary information

Supplementary Information for Scalable optical vortex arrays enabled by the decomposition of Laguerre–Gaussian beams into three Hermite–Gaussian modes and multibeam interference.

## Data Availability

The data that support the findings of this study are available from the corresponding author upon reasonable request.
