# Supplementary material for: Scalable optical vortex arrays enabled by the decomposition of Laguerre–Gaussian beams into three Hermite–Gaussian modes and multibeam interference
Source: Light Sci Appl. 2026 Apr 8;15:193. doi: 10.1038/s41377-026-02254-0 (PMC13062030; doi:10.1038/s41377-026-02254-0)
Supplement: Supplementary file 1 — Supplementary Information for Scalable optical vortex arrays enabled by the decomposition of Laguerre–Gaussian beams into three Hermite–Gaussian modes and multibeam interference. [file 41377_2026_2254_MOESM1_ESM.pdf]

Supplementary Information for Scalable optical vortex arrays enabled by the decomposition of Laguerre–Gaussian beams into three Hermite–Gaussian modes and multibeam interference

Yoshiki Nakata[1]*, Noriaki Miyanaga[2], Yuki Kosaka[3], Masataka Yoshida[4]

[1] Institute of Laser Engineering, The University of Osaka, 2-6 Yamadaoka, Suita, Osaka 565-0871 Japan.

[2] Institute for Laser Technology, 1-8-4 Utsubo-honmachi, Nishi-ku, Osaka, 550-0004 Japan.

[3] JGC Holdings, 2-3-1 Minato Mirai, Nishi-ku, Yokohama-shi, Kanagawa, 220-6001 Japan.

[4] Osaka Gas Co., Ltd., 4-1-2 Hiranomachi, Chuo-ku, Osaka 541-0046 Japan.

Corresponding author: nakata.yoshiki.ile@osaka-u.ac.jp

This file contains:

1. Derivation of the formation of an LG_{01} mode by a set of three HG_{01} modes
2. Triangular array of spots in a six-beam interference pattern and period
3. Spatiotemporal variations in the distribution of electric field
4. Specifications of the DOE and the spiral phase plate

Supplementary Information:

1. Derivation of the formation of an $\mathbf{LG}_{0,1}$ mode from a set of three $\mathbf{HG}_{0,1}$ modes

The combination of the product of the Hermite polynomial $(H_n(x), H_m(y))$ and the relation with the Laguerre polynomial (L_p^l) is as follows[1]:

$$\sum_{k=0}^{n+m}(2i)^k p_k^{(n-k,m-k)}(0)H_{n+m-k}(x)H_k(y) \propto \begin{cases}(-1)^m(x+iy)^{n-m}L_m^{n-m}(r^2) & \text{for } n \geq m \\ (-1)^n(x+iy)^{m-n}L_n^{m-n}(r^2) & \text{for } n < m\end{cases} \quad (1)$$

with

$$p_k^{(n-k,m-k)}(0) = \frac{(-1)^k}{2^k k!}\frac{d^k}{dt^k}[(1-t)^n(1+t)^m]\bigg|_{t=0} \quad (2)$$

where n and m are the numbers of transverse modes in the x and y directions. In the case of $n = 1$ and $m = 0$,

$$(x+iy)\ \ L_0^1(r^2) = H_1(x)H_0(y) + iH_0(x)H_1(y) \quad (3)$$

The Hermite polynomial under a 45° rotation is given by[2]

$$\sum_{k=0}^{n+m}(-2)^k p_k^{(n-k,m-k)}(0)H_{n+m-k}(x)H_k(y) = \left(\sqrt{2}\right)^{n+m} H_n\left(\frac{x-y}{\sqrt{2}}\right)H_m\left(\frac{x+y}{\sqrt{2}}\right) \propto H_n\left(\frac{x-y}{\sqrt{2}}\right)H_m\left(\frac{x+y}{\sqrt{2}}\right) \quad (4)$$

and hence,

$$E_{0,1}^{LG} \propto E_{0,1}^{HG} + e^{i\frac{\pi}{2}} E_{1,0}^{HG} \tag{5}$$

Next, we use the following coordinate rotation formulas:

$$\begin{cases} X' = X\cos\theta - Y\sin\theta \\ Y' = Y\cos\theta + X\sin\theta \end{cases} \tag{6}$$

Equation (4) can be extended for an arbitrary degree rotation system in the $HG_{0,1}$ mode as follows:

$$H_0(x\cos\theta - y\sin\theta)H_1(x\sin\theta + y\cos\theta) \propto H_1(x)H_0(y)\sin\theta + H_0(x)H_1(y)\cos\theta. \tag{7}$$

Here, θ is the angle with respect to the positive direction of the x-axis, with clockwise being positive. The sum of the three electric fields, with a phase difference $e^{i\theta}$, applied to the $HG_{0,1}$ mode that is rotated through an angle $\theta = 0, \pm 60°$ is given by

$$H_0(x)H_1(y) + \frac{\sqrt{3}}{2}\left(\frac{1}{2} + i\frac{\sqrt{3}}{2}\right)H_1(x)H_0(y) + \frac{1}{2}\left(\frac{1}{2} + i\frac{\sqrt{3}}{2}\right)H_0(x)H_1(y)$$

$$- \frac{\sqrt{3}}{2}\left(\frac{1}{2} - i\frac{\sqrt{3}}{2}\right)H_1(x)H_0(y) + \frac{1}{2}\left(\frac{1}{2} - i\frac{\sqrt{3}}{2}\right)H_0(x)H_1(y)$$

$$= \frac{3}{2}\left(H_0(x)H_1(y) + iH_1(x)H_0(y)\right)$$

$$\propto (x + iy)L_0^1(r^2) \quad (\because (3)) \tag{8}$$

and then,

$$E_{0,1}^{\mathrm{LG}} \propto E_{0,1}^{\mathrm{HG}} + e^{i\frac{\pi}{3}} E_{0,1}^{\mathrm{HG}}(60^{\circ}) + e^{-i\frac{\pi}{3}} E_{0,1}^{\mathrm{HG}}(-60^{\circ}) \tag{9}$$

2. Triangular array of spots in a six-beam interference pattern and its period

The spatial light distribution of the OVA_0 mode ($\alpha_n = 0$) with $\phi_1 = 0°$ is expressed as follows[3]:

$$I(x, y) = 4{E_{n0}}^2\pi\left(\cos(2Ax) + 2\cos(Ax)\cos\left(\sqrt{3}Ay\right)\right) \tag{10}$$

where $A = \frac{1}{2}k\sin\theta$. The partial derivatives of this equation with respect to $x\ and\ y$ are as follows:

$$\frac{\partial I}{\partial x} = -16AE_{n0}{}^{2}\pi\left(\sin 2Ax + \sin Ax \cos\sqrt{3}Ay\right) * \left(\cos 2Ax + 2\cos Ax \cos\sqrt{3}Ay\right)$$
$$\frac{\partial I}{\partial y} = -16\sqrt{3}AE_{n0}{}^{2}\pi \cos Ax \sin\sqrt{3}Ay \left(\cos 2Ax + 2\cos Ax \cos\sqrt{3}Ay\right) \tag{11}$$

respectively. First, $\frac{\partial I}{\partial x} = \frac{\partial I}{\partial y} = 0$ at the points where the light intensity reaches its maximum value. Hence,

$$\left.\frac{\partial I}{\partial x}\right|_{x=0} = -16AE_{n0}{}^{2}\pi\left(\sin 0 + \sin 0 \cos\sqrt{3}Ay\right) * \left(\cos 0 + 2\cos 0 \cos\sqrt{3}Ay\right) = 0$$
$$\left.\frac{\partial I}{\partial y}\right|_{x=0} = -16\sqrt{3}AE_{n0}{}^{2}\pi \cos 0 \sin\sqrt{3}Ay \left(\cos 0 + 2\cos 0 \cos\sqrt{3}Ay\right) = 0 \tag{12}$$

Then, the following equation is obtained:

$$y = \frac{n\pi}{\sqrt{3}A}, \frac{\left(2n + 1 \pm \frac{1}{3}\right)\pi}{\sqrt{3}A} \tag{13}$$

where n is a natural number. The coordinates of the centre spot are $(x, y) = (0,0)$. The coordinates of the next spot are $(x, y) = \left(0, \frac{2\pi}{\sqrt{3}A}\right)$. Thus, $\Lambda = \frac{2\pi}{\sqrt{3}A} = \frac{4\pi}{\sqrt{3}k\sin\theta}$ and is constant for any value of ϕ_1. Therefore, the period Λ is expressed by the same equation as for $\phi_1 = 0°$ in the main text. In addition, the points in the OVA_0 mode and the singularities in the OVA_1 mode have the same coordinates, as shown in Figs. 3b and c of the main text.

3. Spatiotemporal variations in the distribution of the electric field

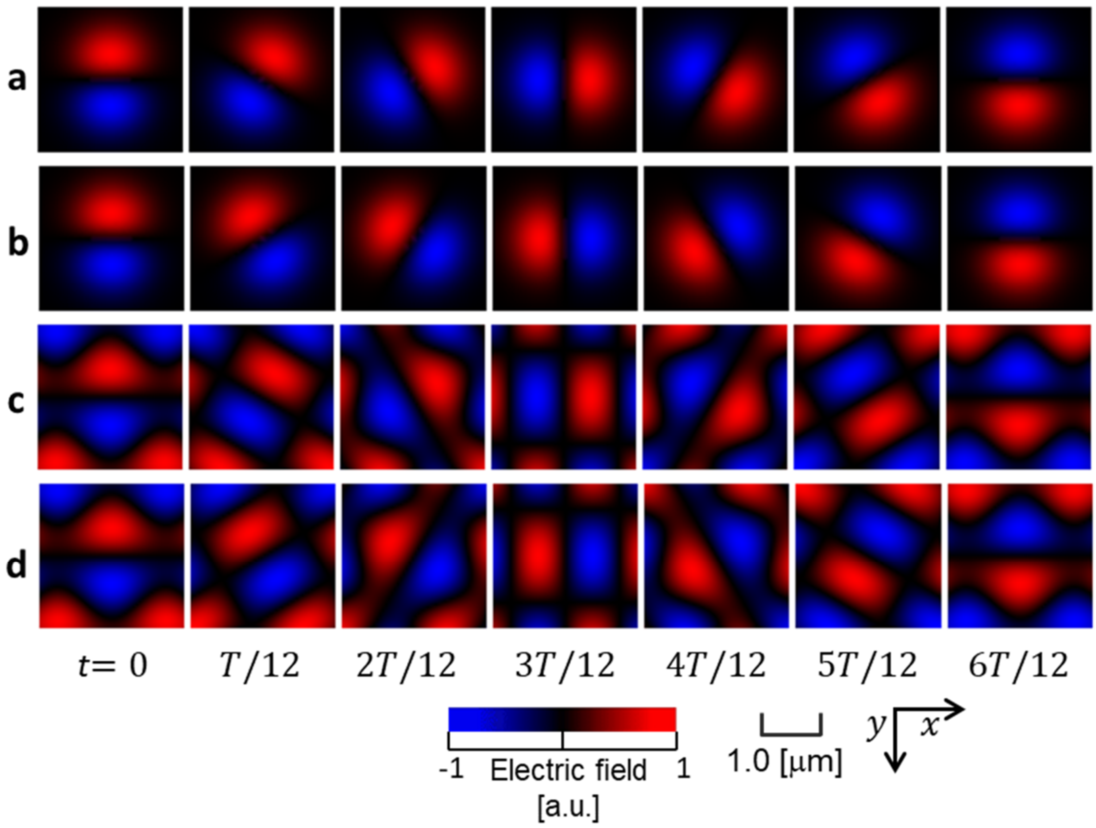

Figure S1. Spatiotemporal variations in electric field distribution: (a) $\mathrm{LG}_{0,1}$ mode, (b) $\mathrm{LG}_{0,-1}$ mode, (c) OVA_1 mode, (d) OVA_{-1} mode. The centre of each image is located at (0,0). Images: numerical simulations (Wolfram Mathematica).

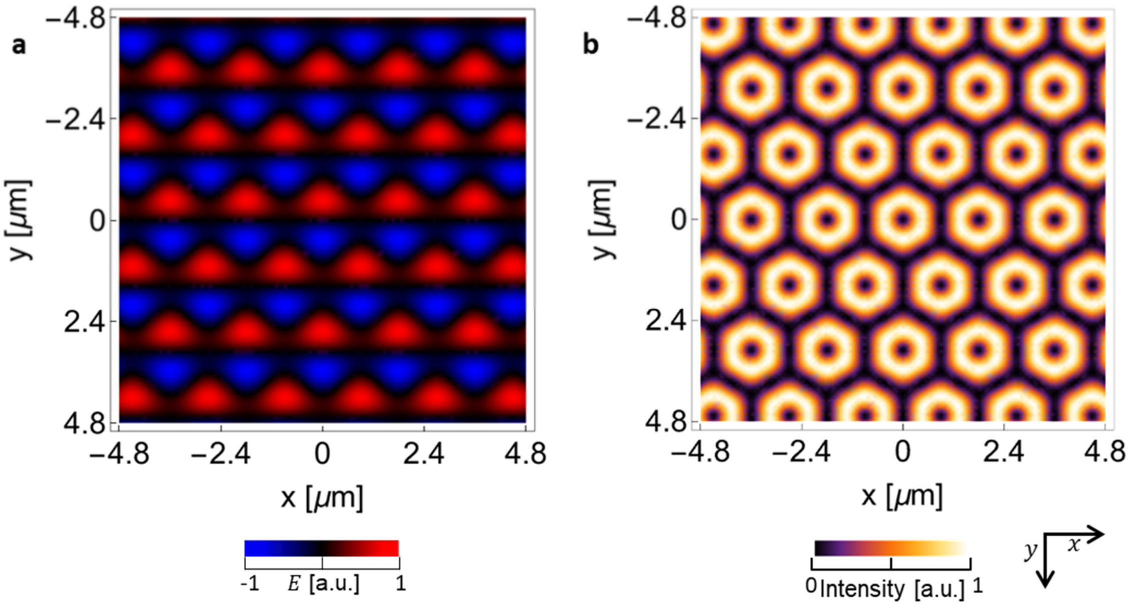

Figure S2. (a) Spatiotemporal distribution in the electric field of the OVA_1 mode at $t = 0$; (b) pattern of OVA_1. These are simulated by equations (5) and (7), respectively.

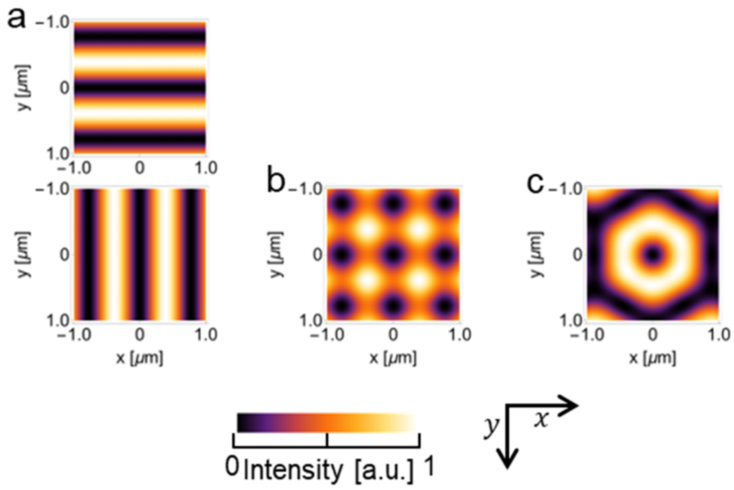

Figure S3. Comparison between two- and three-interference-pattern representations: (a) pairwise two-interference-pattern (2-IP) configuration. (b) four-beam interference ($N = 4$, 2-IP) corresponding to the two-mode HG decomposition ($\Delta\alpha = \pi/2$, $\Delta\phi = 2\pi/N = \pi/2$), which produces four intensity maxima in a square lattice with a single central phase singularity, rather than a circular optical vortex array. (c) Three-interference-pattern (3-IP) representation used in the present six-beam interference scheme, yielding a stable and symmetric optical vortex array.

These results show that the conventional two-mode HG decomposition does not directly map onto a scalable interference framework for optical vortex arrays, whereas the three-mode representation does.

4. Specifications of the DOE and the spiral phase plate

Table S1. Specifications of the DOE (MS609RYX, Holo/Or Ltd.)

Material	Fused silica
Diameter [mm]	25.4
Clear aperture [mm]	22.9
Thickness [mm]	3
Coating	AR/AR coating
Number of spots	6 (diffracted) +1 (zero-order)
Power uniformity	Approximately equal among six beams
Azimuthal spacing	60° increments
Full diffraction angle [deg.]	1.31 at 488 nm, 1.43 at 532 nm
Overall efficiency	~ 77% at 488 nm (manufacturer specification), 61% at 532 nm (measured)

Table S2. Specifications of the spiral phase plate (VL-209-QYA, Holo/Or Ltd.)

Material	Fused silica
Diameter [mm]	25.4
Clear aperture [mm]	22.9
Thickness [mm]	3
Coating	AR/AR coating
Designed wavelength [nm]	532
Topological charge	1

1. Allen, L., Beijersbergen, M. W., Spreeuw, R. J. C. & Woerdman, J. P. Orbital angular momentum of light and the transformation of Laguerre-Gaussian laser modes. *Phys. Rev. A - At. Mol. Opt. Phys.* **45**, 8185–8189 (1992).
2. Abramochkin, E. & Volostnikov, V. Beam transformations and nontransformed beams. *Opt. Commun.* **83**, 123–135 (1991).
3. Yoshida, M. Effects of inter-beam complex amplitude modulation and spin angular momentum. (Master's thesis, Osaka University, 2018).
